# Chromosomal Organization and Subgenomic Differentiation of Satellite Repeats in Hexaploid *Elymus tsukushiensis*

**DOI:** 10.3390/plants15213254

**Published:** 2026-10-22

**Authors:** Viktoria M. Sokolova, Anna I. Yurkina, Daniil S. Ulyanov, Gennady I. Karlov, Mikhail G. Divashuk, Pavel Yu. Kroupin

**Affiliations:** All-Russia Research Institute of Agricultural Biotechnology, Timiryazevskaya St., 42, 127434 Moscow, Russia; vika-kuz367@yandex.ru (V.M.S.); aaaaaa3197@gmail.com (A.I.Y.); uldas1508@gmail.com (D.S.U.); karlov@iab.ac.ru (G.I.K.); divashuk@gmail.com (M.G.D.)

**Keywords:** *Elymus tsukushiensis*, satellite DNA, fluorescence in situ hybridization (FISH), Triticeae, Y genome, cytogenetic markers

## Abstract

The hexaploid grass *Elymus tsukushiensis* (2n = 42, StStHHYY) provides a suitable system for investigating the chromosomal organization and subgenomic differentiation of repetitive DNA in an allopolyploid genome. In this study, we determined the chromosomal localization of 16 previously characterized repetitive families (E1–E16) derived from satellite DNA and used as FISH probes and subsequently identified the H-, St-, and Y-subgenomes with Oligo-HvCSR and St_2_-80 probes. FISH mapping revealed pronounced differences among subgenomes: large arrays of E7 and E9 were associated with the St-subgenome; E10 was detected exclusively on Y-chromosomes; E12 and E16 showed preferential localization in the H-subgenome; the remaining repeats exhibited two- or multisubgenomic distribution. Comparison of FISH patterns with previously established relative copy numbers showed that quantitative abundance and physical organization are not strictly proportional: the high copy number of E9 coincided with local accumulation on one pair of St-chromosomes, whereas the low abundance of E16 did not preclude the formation of reproducible FISH loci primarily in the H-subgenome. Repeatome analysis indicated that repetitive DNA constitutes 50.36% of the *E. tsukushiensis* genome, including 38.42% mobile elements and 1.41% satellite DNA. Our results demonstrate that combining quantitative and cytogenetic approaches provides a more complete characterization of subgenomic organization of satellite repeats and identifies E9, E10, E12, and E16 as promising candidates for further evaluation as cytogenetic markers in Triticeae.

## 1. Introduction

The genus *Elymus* L. *sensu lato* represents one of the most complex allopolyploid groups within the tribe Triticeae. Depending on the taxonomic concept adopted, it comprises about 150–200 species whose genomic organization includes various combinations of the basic haplomes St, H, P, W, and Y. The St- and H-haplomes are phylogenetically related to *Pseudoroegneria* and *Hordeum*, respectively, whereas a diploid representative corresponding to the Y-haplome has not yet been found [1,2,3]. In genome-taxonomic systems, species with the StHY combination are also placed in the genus *Campeiostachys* [4]. The hexaploid *Elymus tsukushiensis* Honda (2n = 42, StStHHYY) belongs to this group and provides a suitable model for investigating the chromosomal organization and subgenomic differentiation of repetitive DNA in a allopolyploid genome [5,6,7].

Repetitive DNA accounts for a substantial portion of Triticeae genomes and is among the most dynamic components of their chromosomes [8,9]. Satellite DNAs—tandemly organized repetitive families—occupy a special place, as they differ considerably in copy number and chromosomal organization. Current views consider satellite DNA as a dynamic system in which the maintenance of homologous repeat families in related genomic lineages coexists with independent amplification, reduction, and structural reorganization of individual arrays [10,11]. Substantial differences in copy number and chromosomal localization of homologous repeat families have been documented in polyploid wheats, in representatives of *Hordeum*, and in closely related St-genome species of *Pseudoroegneria* [12,13,14].

The evolution of satellite DNA may involve several interconnected molecular processes. Changes in copy number and the size of tandem arrays can result from recombination- and replication-associated mechanisms, including unequal crossing-over and replication slippage, whereas gene conversion and other homogenizing processes may contribute to sequence similarity among repeat units within a family. Additional mechanisms, including rolling-circle amplification and associations with transposable elements, may contribute to the amplification, dispersal, and reorganization of satellite repeats [10,11,15]. In an allopolyploid genome, interpretation of these processes is further complicated by the fact that the constituent subgenomes already carry repeat complements that evolved independently before genome merger. Consequently, differences observed among the subgenomes of an extant polyploid may reflect either variation inherited from ancestral genomic lineages or subsequent changes in repeat abundance and chromosomal organization following allopolyploidization [13,16]. Therefore, differences in copy number or FISH patterns alone do not allow the underlying evolutionary mechanism or the timing of the observed changes to be determined unambiguously.

Repetitive sequences are widely used to investigate genomic differentiation in *Elymus*. Physical mapping of Afa-like, AAG-, and ribosomal repeats revealed differences among the St-, H-, and Y-subgenomes [17], and the development of FISH approaches has enabled the construction of molecular karyotypes for several *Elymus* species, the differentiation of individual chromosomes, and the detection of intra- and intergenomic rearrangements [18,19,20]. Nevertheless, systematic comparison of the quantitative representation of a large set of satellite families with their chromosomal localization in a single StHY genome remains insufficiently explored.

In addition, a cytogenetic pattern observed in a single plant or accession should not necessarily be regarded as a fixed species-level characteristic. Pronounced intra- and interpopulation variation in FISH patterns has been reported in *Elymus nutans* Griseb., including variation in repetitive-sequence abundance and chromosome structure [21,22]. Intraspecific variation in the chromosomal distribution of tandem repeats has also been demonstrated among accessions of the St-genome species *Pseudoroegneria libanotica* Hack [23]. Thus, reproducibility of a FISH pattern within a studied genotype, its stability across accessions or populations, and its inheritance across generations represent distinct levels of evidence and should be evaluated separately. Reproducibility among metaphase cells of the same individual demonstrates consistency within the analyzed somatic material but does not by itself establish stable transmission of the pattern across generations. Cytogenetic stability across generations can instead be evaluated by analysis of progeny and/or meiotic chromosome transmission [24]. Accordingly, assessment of the broader stability and diagnostic value of a repeat pattern requires biologically independent and representative sampling, including multiple plants and accessions and, where relevant, material from different populations or geographic origins. Such sampling is necessary to distinguish individual- or accession-specific variation from patterns that are consistently associated with a species or subgenome.

The Y-haplome is of particular interest because its origin remains one of the debated issues in *Elymus* evolution [25]. Molecular cytogenetic data reveal pronounced similarity of certain repetitive DNA components between the St- and Y-subgenomes [26], whereas the chromosome-level assembly of *E. nutans*. Placed Y as sister to the V/J^v^ clade represented by genomic lines of *Dasypyrum* and *Thinopyrum* [1]. These findings further motivate the analysis of subgenomic organization of repetitive DNA in StHY genomes.

In our previous work, comparative analysis of repeats E1–E16 across Triticeae representatives allowed us to characterize their interspecific copy-number variation and to identify families with varying degrees of association with particular genomic components [27]. However, quantitative data alone cannot establish whether such association corresponds to preferential localization of the main repeat arrays in a specific subgenome or reflects other aspects of its overall genomic abundance. This raises the hypothesis that quantitative repeat abundance and chromosomal localization represent related but non-equivalent characteristics of satellite DNA organization in a polyploid genome.

We hypothesized that the E1–E16 satellite repeat families differ in their chromosomal organization among the St-, H-, and Y-subgenomes of *E. tsukushiensis* and that their whole-genome quantitative abundance does not necessarily predict the subgenomic localization of their major chromosomal arrays. The aim of the present study was therefore to characterize the chromosomal organization and subgenomic differentiation of these repeats in *E. tsukushiensis*. To address this aim, we (i) characterized the repeatome of *E. tsukushiensis*; (ii) determined the chromosomal localization of E1–E16 by FISH and assigned the corresponding chromosomes to the St-, H-, and Y-subgenomes; (iii) compared the resulting FISH patterns with previously determined relative copy-number data; and (iv) evaluated the potential of individual repeat families for further development as subgenome-associated cytogenetic markers.

## 2. Results

### 2.1. Repeatome Structure of E. tsukushiensis

The detailed distribution of the major classes and lineages of repetitive sequences is presented in Table 1. The total proportion of repetitive sequences in the *E. tsukushiensis* genome was 50.36%.

Mobile genetic elements accounted for the major fraction of the repeatome (38.42%), predominantly represented by Class I retrotransposons (36.87%), among which the Ty3/Gypsy and Ty1/Copia superfamilies were the most abundant. Satellite repeats accounted for 1.41% of the genome, whereas 45S and 5S ribosomal DNA represented 0.37% and 0.06%, respectively. Unclassified and other repetitive sequences accounted for 10.10% of the genome. Among retrotransposons, elements of the Ty3/Gypsy superfamily predominated, whereas Ty1/Copia elements represented a smaller fraction of the repeatome. Among Class II DNA transposons, EnSpm/CACTA elements were the most abundant.

The E1–E16 repeats investigated in this study represent 16 individual satellite DNA families belonging to the satellite repeat category presented in Table 1. These repeats were previously identified and characterized in our earlier study [27] and were selected here for detailed chromosomal localization by FISH.

### 2.2. Identification of Subgenomes in E. tsukushiensis

Subgenome identification was performed by comparing the obtained hybridization patterns with published data on the chromosomal localization of Oligo-HvCSR and St_2_-80 probes. For the H-subgenome, we relied on the results of [19], which showed that Oligo-HvCSR predominantly marks centromeric regions of H-subgenome chromosomes in *Elymus dahuricus* Turcz. The authors also observed an additional pair of chromosomes with Oligo-HvCSR signals, which they attributed to possible redistribution of the corresponding repeat between the H- and St-subgenomes. For differentiation of St- and Y-subgenomes, we considered St_2_-80 as a marker of the St genome and its application in cytogenetic characterization of StY-containing Triticeae species [18,28].

Based on these data, chromosomes were assigned to subgenomes according to the combined hybridization patterns of the two probes. Chromosomes showing prominent centromeric Oligo-HvCSR signals and weak dispersed St_2_-80 signals were assigned to the H-subgenome. Chromosomes exhibiting intense St_2_-80 hybridization signals distributed along the chromosomes, with more prominent clusters particularly in the subtelomeric regions, were assigned to the St-subgenome; the presence of an additional Oligo-HvCSR signal on one pair of such chromosomes was considered as variability in the distribution of this repeat, as previously described in *Elymus* [19]. Chromosomes lacking the characteristic centromeric Oligo-HvCSR signals and differing from St-chromosomes by the absence of intense St_2_-80 signals, including prominent clusters in the subtelomeric regions, while showing only weak dispersed signals, mainly in terminal regions—were assigned to the Y-subgenome.

Thus, the correspondence of the observed hybridization patterns to the previously described localization of these probes served as the basis for assigning the chromosomes of *E. tsukushiensis* to the H-, St-, and Y-subgenomes (Figure 1).

### 2.3. FISH Localization of Satellite Repeat Clusters E1–E16 on Chromosomes of E. tsukushiensis

FISH mapping of the repetitive DNA sequences E1–E16, previously identified by us in representatives of *Elymus* and *Elytrigia*, revealed differences in their chromosomal localization. The investigated repeats formed both dispersed and localized signals and varied in subgenomic distribution, number and position of hybridization sites, and signal intensity (Figure 2, Figure 3 and Figure 4).

Cluster E13 is dispersed in interstitial regions of both arms of all chromosomes (Figure 2(A1–A3)). Sequence analysis showed similarity (Table S1) to the terminal and/or pericentromeric satellites StLIB96 and StLIB117 described in *P*. *libanotica* (St genome) [23]; to the terminal tandem repeat pSc250 in *Secale cereale* L. (R genome) [29]; the terminal microsatellite StY_90_c y *Roegneria ciliaris* Trin. and *Roegneria grandis* Keng. (StY гeнoм) [18]; and to the pericentromeric satellites CentT550 and CL261, which localize predominantly on the D genome of *Aegilops tauschii* Coss., *Aegilops crassa* Boiss., and *Triticum aestivum* L. [30,31].

Cluster E11 produces terminal signals on the 13 pairs of chromosomes, predominantly on short arms (Figure 2(A1–A3)). Signals are detected on three pairs of H-subgenome chromosomes and four pairs of St-subgenome chromosomes; one St-pair additionally shows an interstitial signal on the long arm. On six Y-subgenome pairs, signals are located on short arms, with one of these pairs also showing an additional terminal signal on the long arm. E11 shows similarity to the terminal StY_90_c described in R. Ciliaris and R. Grandis (StY genome) [18], and to the satellite Lt1-6 from *Leymus triticoides* (Buckley) (Ns genome) [32].

E15 is a dispersed repeat present on all chromosomes, predominantly in terminal regions (Figure 2(B1–B3)). The most intense signals occur on H-subgenome chromosomes. No homologous sequences with previously established chromosomal localization were found.

Cluster E14 is localized on St- and Y-subgenome chromosomes (Figure 2(B1–B3)). In St-subgenome chromosomes, the signal distribution pattern is as follows: two pairs show pericentromeric signals; two other pairs exhibit proximal signals on both arms in addition to pericentromeric signals; one pair displays a distal signal on the long arm. Among Y-subgenome chromosomes, two pairs show pericentromeric signals; one pair combines pericentromeric and terminal signals on the long arm; another pair shows distal signals on the long arm, and one pair terminal signals on the short arm. Sequence analysis of E14 revealed similarity to repetitive sequences with terminal and/or pericentromeric localization in *Pseudoroegneria* [23], *S. cereale* [29], *R. grandis* [18], *Ae. crassa*, *Ae. tauschii* and *T. aestivum* [30,31].

E10 produces signals exclusively on Y-subgenome chromosomes (Figure 2(C1–C3)): three pairs show proximal and interstitial signals on both arms, with two of these also having pericentromeric signals; one pair has an interstitial signal on the long arm, and another has proximal signals on the long arm. This cluster showed similarity to microsatellite P523, which gives pericentromeric signals on J^s^-subgenome chromosomes of *Thinopyrum intermedium* (Host) Barkworth & D.R. Dewey [33] and to a satellite with terminal localization on J^b^-genome chromosomes of *Thinopyrum bessarabicum* Săvul. & Rayss [31].

E12—a terminally localized cluster—shows the strongest signals on the H-subgenome of *E. tsukushiensis* (Figure 2(C1–C3)). Signal intensity varies among chromosomes: three pairs display the strongest signals, four pairs moderate signals, and two pairs the weakest signals. Among H-subgenome chromosomes, one pair shows terminal signals on both arms; another pair also has signals on both arms but with a weak proximal signal instead of a terminal one on the long arm. Two pairs have terminal signals on the short arm, and three pairs on the long arm. Among St-chromosomes, only one pair shows a terminal signal on the short arm, and among Y-chromosomes one pair has a terminal signal on the long arm. We found similarity of E12 to repetitive sequences of *Hordeum vulgare* L. (H genome) with terminal and subtelomeric localization [34,35].

Cluster E9 shows an intense proximal signal on the short arm of one pair of St-subgenome chromosomes (Figure 3(A1–A3)). Sequence analysis revealed similarity to satellite CL232, which exhibits terminal and/or distal localization in common wheat (A and D subgenomes), *Ae. tauschii*, and *Ae. crassa* (on D and X^cr^ subgenomes of the tetraploid form and on D1, D2, and X^cr^ of the hexaploid) [31].

Cluster E16 is localized on six pairs of H-subgenome chromosomes (Figure 3(A1–A3)). On five pairs, signals are predominantly detected in distal regions of the short or long arm, whereas one pair shows a proximal signal on the short arm. One H-pair exhibits the most intense hybridization signal. In addition, a proximal signal on the short arm is observed on one pair of Y-subgenome chromosomes. Homology analysis did not reveal any known repetitive sequences similar to cluster E16.

E2 is localized terminally on the long arm of two pairs of H-chromosomes, and distally on the long arm of another H-pair (Figure 3(B1–B3)). On St-chromosomes, the labeling pattern is: distal on the short arm of one pair, interstitial on the long arm of a second pair, and a weak pericentromeric signal on a third pair. Cluster E2 is similar to FISH sequences of common wheat that show terminal, distal, and interstitial signals on the A and B subgenomes [36].

Cluster E4 displays numerous signals on all H-subgenome chromosomes, located in pericentromeric, proximal, interstitial, distal, subterminal, and terminal regions (Figure 3(B1–B3)). On St-chromosomes, the repeat is detected on five pairs, mainly in subterminal and terminal regions. Two pairs show the most intense terminal-distal signals on the short arm, and one of these additionally has a proximal signal on the short arm. Y-subgenome chromosomes exhibit a different pattern: one pair combines pericentromeric and distal signals on the long arm, a second has distal signals on the long arm, and a third has distal signals on the short arm. Sequence analysis showed that E4 is similar to pAs1-like repetitive sequences CL3 from *Ae. crassa* [31], pTa-173 from *T. aestivum* [36], and to microsatellite P720, which localizes terminally and interstitially on V, St, J^b^, J^vs^, J^r^, A, B, and D genomes [33,37,38].

Sequence E3 is localized distally, proximally, interstitially, and terminally (Figure 3(C1–C3)). On H-subgenome chromosomes, E3 signals are detected on three pairs: one pair shows the most intense signals on the short arm, while the other two have distal signals on the short or long arm. The strongest hybridization of E3 is observed on St-chromosomes: one pair displays extended diffuse signals in the proximal-interstitial region of the short arm; three pairs show distal signals on both arms; another pair has proximal and terminal signals on the long arm. On Y-chromosomes, E3 signals are generally weaker than on St and fewer in number: one pair shows proximal signals on the short arm and weak interstitial and terminal signals on the long arm; another pair has distal signals on the short arm; a third pair shows interstitial signals on the long arm and distal signals on the short arm. Homology analysis indicated that E3 is similar to satellite CL148, which has predominantly distal localization on J^b^-genome chromosomes of *Thinopyrum* [31], and to ACRI_TR_CL80 with pericentromeric and subtelomeric localization on the P genome of *A. cristatum* (L.) Gaertn [39].

Sequence E8 is characterized mainly by subterminal, proximal, and distal signals (Figure 3(C1–C31)). On one pair of H-chromosomes, the E8 signal is subterminal on the long arm. The strongest hybridization is observed on one pair of St-chromosomes, with a diffuse signal in the proximal region of the short arm. In addition, another St-pair shows weak distal signals on the short arm. In terms of chromosomal localization, cluster E8 resembles several previously characterized repeats, including the subtelomeric repeat Spelt52.2 found in S, A^t^, and G genomes [40,41], satellite CL244 with terminal localization in J^b^, X^cr^, and B genomes [31]; and the terminal satellite Lt1-6 described for the N^s^ genome [32].

Cluster E6 is detected on three pairs of H-subgenome chromosomes, four pairs of St-chromosomes, and two pairs of Y-chromosomes (Figure 4(A1–A3)). In the H-subgenome, two pairs show intense terminal signals: one on the short arm, the other on the long arm; a third pair exhibits a weaker subterminal signal. In the St-subgenome, two pairs have intense terminal signals on short arms, and two pairs on long arms. In the Y-subgenome, one pair shows terminal signals on both arms, and another pair has a terminal signal on the short arm. Cluster E6 shows similarity to previously described repetitive sequences with predominantly terminal and subterminal localization: subterminal signals were found in *Ae. crassa* and *Th. bessarabicum* [31], and terminal signals in *P. libanotica* and *Pseudoroegneria tauri* (Boiss. & Balansa) Á.Löve [14], as well as in the S genome of *Ae. speltoides* Tausch and the B/G genomes of polyploid wheats. In the S genome of other diploid and polyploid *Aegilops* species, corresponding signals are absent [42,43,44].

Cluster E1 is detected on six pairs of H-subgenome chromosomes (Figure 4(A1–A3)). On four pairs, signals are distal on the short arm, with one of them showing an intense signal. On one pair, the strongest signals are subterminal on both arms. On other chromosomes, signals are mostly weak and minor; for example, one pair shows a dispersed signal in the distal region of the long arm. In the St-subgenome, the strongest E1 signals are found on two pairs in the subterminal region of the short arm, while less intense dispersed signals are observed on the long arm of these chromosomes. Two other pairs show distal signals on the short arm, and two pairs have weak distal signals on the long arm. In the Y-subgenome, E1 displays intense and clearly localized signals on three pairs: on two pairs they are proximal on the short arm, whereas on the third pair both proximal and subterminal signals are present on the short arm. Additionally, two other pairs show distal signals on the short or long arm. Homologs of cluster E1 are characterized by predominantly distal and subterminal localization. In *P. libanotica* and *P. tauri*, weak terminal and distal signals are observed in the St genome [14], in *Th. bessarabicum*, interstitial signals in the J^b^ genome. In *Ae. crassa* and *Ae. tauschii*, the most intense signals are distal/subterminal in the D genome [31]. In *T. aestivum*, the repeat is predominantly distal in A-, B-, and D-genomes, with abundance decreasing in the order A > B > D [36].

Cluster E5 produces intense interstitial signals on the short arm of one St-pair, and weaker proximal-pericentromeric signals on the short arm of two other St-pairs (Figure 4(B1–B3)). In the Y-subgenome, the cluster localizes proximally-pericentromerically on the long arm of two pairs and interstitially on the long arm of one pair. Homologs of E5 show predominantly pericentromeric localization in *P. libanotica* and *P. tauri*, with additional weak interstitial signals in *P. libanotica* [14].

E7 is localized only on two pairs of St-subgenome chromosomes (Figure 4(B1–B3)): one pair shows only pericentromeric signals; the second pair, in addition to pericentromeric signals, displays the most intense interstitial signals on both arms. Homologs of E7 are characterized by predominantly pericentromeric localization in *P. libanotica* and *P. tauri*, with additional weak interstitial signals in *P. libanotica* [14]. In *Th. bessarabicum*, the homolog is more evenly distributed along chromosomes, with increased representation in distal regions [31].

Overall, FISH mapping revealed a wide spectrum of chromosomal organization among the investigated repeats—from large arrays localized predominantly or exclusively in one subgenome to distribution across two or all three subgenomes. The most pronounced subgenomic association was observed for E7 and E9 (St-subgenome), E10 (exclusively on Y-chromosomes), and E12 and E16 (preferential H-subgenome localization). The remaining repeats showed either two-subgenome (E2, E5, E8, E14) or multisubgenome (E13, E11, E6, E1, E3, E4, E15) distribution.

### 2.4. Comparative Abundance and Chromosomal Distribution of Repeats E1–E16

To compare the FISH mapping results with our previously obtained relative copy number data for E1–E16 [27], we compared the relative copy numbers of these repeats in *E. tsukushiensis* with those in selected diploid species representing the St- and H-genomes—*P. spicata*, *P. tauri*, and *H. vulgare* (Table 2 and Table S2). For comparison, we used log_10_-transformed relative copy number values previously determined by qPCR.

The log_10_ relative copy numbers in *E. tsukushiensis* ranged from −0.2 (E16) to 5.8 (E4). High abundance was also characteristic of E6 (5.0), E11 (4.5), E9 (4.2), E12 (4.2), and E2 (4.1), while E8 (0.7) and E16 (−0.2) had the lowest values.

For some repeats, the quantitative values in *E. tsukushiensis* were close to those of the selected diploid representatives. For example, the copy number of E7 in *E. tsukushiensis* (3.6) was comparable to that in *P. spicata* (3.6) and *P. tauri* (3.2), whereas it was −0.3 in *H. vulgare*. The low abundance of E16 was characteristic of all studied genomes, from −0.7 in *H. vulgare* to −0.2 in *E. tsukushiensis.* For several other repeats, *E. tsukushiensis* showed increased copy numbers relative to all three diploid representatives. The most pronounced difference was for E9, with a value of 4.2 compared to 2.0 and 2.4 in *P. spicata* and *P. tauri*, and −1.0 in *H. vulgare*. For E10, the corresponding value was 3.5, with a range of 1.2–1.8 in diploids; higher abundance in *E. tsukushiensis* was also observed for E4 and E12 (Table 2 and Table S2).

Comparison of quantitative data with FISH mapping revealed various relationships between overall abundance and chromosomal organization. The most illustrative cases are E9, whose high copy number coincided with localization of a large array on one pair of St-chromosomes; E16, whose low abundance did not preclude the formation of reproducible FISH loci mainly in the H-subgenome; and E10, for which the interspecific quantitative association did not match the subgenomic localization of the major arrays (Table 2 and Table S2).

## 3. Discussion

The repeatome of *E. tsukushiensis* W6 PI 283170 was dominated by retrotransposons, particularly Ty3/Gypsy and Ty1/Copia, consistent with previous studies of Triticeae [9,14,40,41,42]. Satellite DNA constituted a relatively small fraction of the genome (1.41%), yet individual satellite families showed pronounced chromosomal differentiation. This contrast indicates that the evolutionary significance of a repetitive DNA family is not determined solely by its contribution to total genome size, because quantitatively minor components may nevertheless undergo pronounced chromosome- and subgenome-specific differentiation.

The estimated repetitive DNA content of *E. tsukushiensis* (50.36%) is considerably lower than the values reported for the chromosome-level genome assemblies of *E. sibiricus* (82.49%) and *E. nutans* (83.89%) [45,46]. This discrepancy may partly reflect differences in repeat-identification approaches, as our analysis was based on low-coverage short-read sequencing and RepeatExplorer2 clustering rather than annotation of a complete genome assembly. Although RepeatExplorer2 provides an effective approach for repeatome characterization, low-abundance or highly divergent repeats may remain undetected or incompletely classified. In addition, unclassified and other repetitive sequences accounted for 10.10% of the genome, indicating that a substantial proportion of the identified repetitive fraction remains insufficiently characterized. Therefore, the reported value should be interpreted as a pipeline-dependent estimate of repetitive DNA content rather than an exhaustive assessment of the genomic repetitive fraction.

When interpreting quantitative data, we consider *P. spicata*, *P. tauri,* and *H. vulgare* as representatives of the respective St- and H-genomic lineages, not as direct progenitors of the *E. tsukushiensis* subgenomes. This caveat is particularly relevant for the H-component, given the complex evolutionary history of H-genomic lineages in *Elymus* [47]. Therefore, comparisons with these diploid representatives provide information on interspecific variation in repeat abundance but do not allow the reconstruction of ancestral repeat organization or the identification of changes specifically associated with allopolyploidization.

### 3.1. Quantitative Representation and Chromosomal Localization as Complementary Characteristics of Satellite Repeats

Comparison of quantitative data with FISH patterns shows that the overall abundance of satellites and their chromosomal organization reflect complementary aspects of repetitive DNA evolution. qPCR-based relative copy number assessment characterizes the total representation of amplifiable copies of the target repeat in the genome, irrespective of chromosomal location, whereas FISH reveals sites of local accumulation and defines their position on individual chromosomes [10,11,48]. FISH signal intensity is influenced not only by genomic copy number but also by tandem organization, local repeat density, sequence homogeneity, and probe accessibility. Consequently, highly abundant repeats may be concentrated in a few chromosomal loci, whereas low-abundance repeats may still form distinct FISH-detectable arrays. Moreover, absence of a detectable FISH signal should not be interpreted as complete absence of the corresponding sequence in a given subgenome, as individual copies or small clusters may fall below the cytological detection threshold. Somatic variation in repeat copy number or chromosomal organization may also contribute to differences in FISH patterns among cells or tissues. However, the present study was restricted to root meristematic cells, and potential tissue-specific or developmental variation in the distribution of E1–E16 repeats was not investigated.

For E7, the relative copy numbers in the St-representatives were considerably higher than in *H. vulgare* (Table 2), and large FISH-detectable arrays were found exclusively on two pairs of St-chromosomes in *E. tsukushiensis*. This result agrees with the previously established association of E7 with St/StY-containing genomic combinations and shows that its major arrays are concentrated in the St-subgenome of the studied accession. Related *Pseudoroegneria* species also show substantial interspecific differences in satellite repeat abundance and chromosomal localization [14,49]. In this comparative context, the St-associated organization of E7 in *E. tsukushiensis* is compatible with retention of an St-lineage-associated repeat component, although subsequent changes in array size or distribution after allopolyploidization cannot be excluded.

An even more pronounced local concentration is observed for E9. Its relative copy number in *E. tsukushiensis* was substantially higher than in the selected diploid representatives (Table 2), whereas intense FISH signals were localized on only one pair of St-chromosomes. This demonstrates that high overall repeat abundance may be associated with local accumulation in a limited number of chromosomal loci. Similar discrepancies between overall repeat abundance and the number of chromosomal loci have been described in the bread wheat satellitome [12]. The occurrence of a comparable relationship in bread wheat suggests that concentration of a highly abundant satellite family within a limited number of major arrays may represent a recurrent mode of satellite DNA organization in polyploid Triticeae rather than a feature specific to E. tsukushiensis. Such patterns are consistent with family-specific changes in the size and organization of tandem arrays during the evolution of polyploid genomes [50].

A different organizational type is exemplified by E4, which exhibits high copy number and widespread chromosomal distribution across all three subgenomes (Table 2). Thus, whereas E9 is concentrated in a single St-chromosome pair, E4 is distributed across numerous chromosomal loci. Comparable variability in the relationship between repeat abundance and chromosomal distribution has been documented in polyploid wheats [13]. Together, the contrasting patterns of E9 and E4 indicate that satellite families within the same polyploid genome may follow different evolutionary trajectories: increased representation may be associated either with localized enlargement of particular arrays or with broader distribution across chromosomes.

### 3.2. Shared Satellite DNA Library and Subgenomic Differentiation

The observed diversity in chromosomal organization of E1–E16 is consistent with the view of satellite DNA as a dynamic genome component, in which related genomic lineages may retain similar repeat families but differ substantially in their abundance and arrangement of chromosomal arrays [51,52]. Evolutionary diversification of satellite families can involve changes in copy number, structure, and genomic distribution, with individual components of the common repeat library evolving at different rates [53,54]. In the studied *E. tsukushiensis* accession, this is reflected in the broad spectrum of organizational patterns—from multisubgenomic distribution to preferential or exclusive localization of large arrays in one subgenome.

Polyploidization provides an additional context for such differentiation, as it brings together previously independently evolving genomes within a single nucleus [16]. In polyploid Triticeae, substantial changes in satellite repeat abundance and chromosomal organization have been demonstrated, for example, in comparisons of bread and durum wheat satellitomes [13]. The observed differences among St-, H-, and Y-components of *E. tsukushiensis* may reflect either divergence inherited from the ancestral genomes or subsequent changes in repeat abundance and chromosomal organization following allopolyploidization. However, the present analysis of a single extant accession does not allow us to distinguish between these scenarios or determine the timing and direction of such changes.

Such heterogeneity of the repetitive fraction has also been observed in other *Elymus* species. Comparative FISH/GISH analysis of six species revealed significant differences among H-, St-, and Y-subgenomes in the distribution of Afa-like, AAG-, and ribosomal repeats, and also uncovered intergenomic rearrangements between H- and Y-chromosomes [17]. In natural populations of the closely related StHY species *E. nutans*, pronounced intraspecific variation in FISH patterns was found, associated with amplification and reduction of sequences; St- and Y-chromosomes were particularly variable [21,22]. Together with the patterns observed in *E. tsukushiensis*, these data indicate that differentiation of repetitive DNA is a recurrent but non-uniform feature of StHY genomes. Importantly, the specific chromosomal patterns differ among species and even among populations, suggesting that repetitive-DNA evolution proceeds independently for different repeat families and genomic lineages rather than through a uniform genome-wide response.

E2 and E14 illustrate partial subgenomic differentiation: the major E2 arrays occur in the H- and St-subgenomes, whereas E14 arrays are distributed between the St- and Y-subgenomes. The presence of related sequences in other Triticeae taxa indicates a broader distribution of these repeat families (Table S1). Therefore, the presence of the same repeat family in St- and Y-subgenomes alone does not provide sufficient evidence for a direct St-origin of Y.

More broadly, comparison with other Triticeae suggests that subgenomic differentiation of satellite DNA is family-specific rather than uniform across the repetitive fraction. Some repeat families remain broadly shared among related genomic lineages, whereas others undergo pronounced changes in abundance or chromosomal distribution. Similar mosaic patterns have been reported in polyploid wheat and different *Elymus* species [13,17,21,22]. Thus, the St-, H-, and Y-subgenomes of *E. tsukushiensis* are unlikely to have undergone a single common trajectory of repetitive-DNA evolution following genome merger; rather, individual satellite families may retain different proportions of ancestral organization and subsequent lineage- or chromosome-specific change.

### 3.3. E10 as a Y-Associated Repeat and a Source of Information on Y-Haplome Organization

E10 shows relatively low abundance in the selected diploid representatives but a higher relative copy number in *E. tsukushiensis* (Table 2), where its major FISH-detectable arrays are localized exclusively on five pairs of Y-subgenome chromosomes. Thus, E10 shows Y-subgenome-specific localization in the studied accession.

Previous qPCR analysis revealed a statistical association of E10 copy number with H-containing genomic combinations [27], whereas FISH mapping localized its major detectable arrays exclusively to the Y-subgenome in the studied *E. tsukushiensis* accession. This discrepancy reflects the different information provided by quantitative analysis and physical mapping and highlights the importance of combining these approaches.

Previous studies based on single-copy nuclear genes suggested distinct origins of the St- and Y-genomes and indicated possible relationships between the Y-genome and other Triticeae genomic lineages [47,55,56]. Chromosome-specific painting revealed similarities between certain repetitive DNA components of the St- and Y-subgenomes [26], whereas the chromosome-level genome assembly of *E. nutans* placed the Y-haplome as sister to the V/J^v^ clade represented by *Dasypyrum* and *Thinopyrum* lines [1].

In this context, the similarity of E10 to P523-related sequences detected in *Thinopyrum* [33], together with its Y-subgenome localization in *E. tsukushiensis,* may reflect the retention of related repeat sequences in the Y- and V/J^v^-related lineages. However, the high evolutionary dynamics of satellite DNA and the possibility of independent amplification or reduction in homologous repeats preclude using the distribution of a single repeat as independent evidence of phylogenetic relationships [51]. This illustrates a more general limitation of satellite DNA as an evolutionary marker: individual repeat families may retain information on historical genomic relationships, but their rapid and lineage-specific turnover can also obscure such relationships. Consequently, evolutionary interpretation is strongest when repeat-distribution data are considered together with independent chromosome-level and sequence-based evidence.

E10 may therefore be considered a potential cytogenetic marker associated with the Y-subgenome in the studied *E. tsukushiensis* accession. However, it cannot serve as a universal marker for all Y-chromosomes, as detectable arrays are present on only five of the seven Y-chromosome pairs. In combination with Oligo-HvCSR and St2-80, E10 may improve the identification of individual Y-chromosomes carrying its major arrays. Further validation across multiple *E. tsukushiensis* accessions and related Triticeae taxa is required to assess its broader applicability as a cytogenetic marker.

### 3.4. Differences in Chromosomal Organization of H-Associated Repeats

Several repetitive DNA families showed preferential association with the H-subgenome, yet their chromosomal organization differed substantially. E12 exhibited similar relative copy numbers in the selected diploid representatives but higher abundance in *E. tsukushiensis* (Table 2). Its major FISH-detectable arrays were preferentially localized in the H-subgenome, despite the presence of weaker signals on St- and Y-chromosomes. The preferential H-association of E12 is consistent with its similarity to *Hordeum*-related repetitive sequences, whereas its presence on St- and Y-chromosomes indicates that the family itself is not restricted to the H lineage. This pattern is therefore more consistent with differential retention and local accumulation of a broadly distributed repeat family than with strict H-specific origin. A fundamentally different case is presented by E16. Despite its extremely low overall abundance in all studied genomes (Table 2), FISH revealed reproducible loci predominantly on H-chromosomes. Comparable low-abundance satellite families forming distinct FISH-detectable loci have also been reported in bread wheat [12]. Thus, E16 illustrates that evolutionary and cytogenetic significance is not determined solely by genome-wide copy number: quantitatively minor repeat families may become locally organized at specific chromosomal sites and thereby contribute disproportionately to chromosome differentiation.

E15 represents another mode of subgenomic differentiation. Unlike E16, it exhibits relatively high copy number and widespread chromosomal distribution across all three subgenomes (Table 2), with the strongest FISH signals observed on H-chromosomes. The similarity of E15 to transposon-derived sequences is particularly relevant in an evolutionary context because associations between satellite DNA and mobile elements have also been described in other Triticeae, including *Aegilops* and *Hordeum* [57,58]. Such examples support the possibility that interactions between different classes of repetitive DNA contribute to the origin, dispersal, or reorganization of satellite arrays and thereby to diversification of repeat landscapes among related species.

Thus, H-association of the studied repeats is realized in at least three distinct ways: preferential accumulation of large arrays with relatively high overall abundance (E12), maintenance of a limited number of organized loci despite low copy number (E16), and relative signal enhancement in the H-subgenome against a background of broad multisubgenomic distribution (E15). This shows that subgenomic differentiation of satellite repeats is not reducible to a single mode of copy-number change but can manifest through different combinations of overall abundance and local chromosomal organization. Such contrasting patterns further support a family-specific model of satellite DNA evolution, in which individual repeats may respond differently to the evolutionary history of the same polyploid genome. Thus, E12 and E16 should be considered preferentially H-subgenome-associated repeats rather than strictly H-subgenome-specific markers, as their signals are also detected on chromosomes of other subgenomes.

### 3.5. Widely Distributed Repeats and Their Cytogenetic Informativeness

Among the studied repeats, E13 showed the broadest chromosomal representation: dispersed interstitial signals are detected on all H-, St-, and Y-chromosomes. Together with its relatively moderate quantitative variation among the surveyed genomic lineages (Table 2), this broad chromosomal distribution suggests greater conservation of E13 than of repeats showing pronounced subgenome-associated accumulation. Such widely distributed families may represent components of a shared repeat repertoire retained across the genomic lineages combined in the polyploid genome.

E11 displayed a different pattern. In *E. tsukushiensis*, its signals are found on 13 chromosome pairs—three H-, four St-, and six Y-pairs—whereas quantitative representation varied substantially among the surveyed diploid representatives. Nonetheless, in our previous study E11 proved informative for labeling chromosome 4VL of *Dasypyrum villosum* in a wheat background [27]. A similar principle is known for other widely distributed Triticeae repeats, such as GAA- and Afa-like sequences, which are used to identify individual chromosomes and genomic components due to their specific hybridization patterns [59].

A similar trend is seen for E6. In *E. tsukushiensis,* this repeat is detected on three H-, four St-, and two Y-chromosome pairs and thus has broad subgenomic representation. Moreover, in our previous study E6 proved effective as a marker for V-chromosomes in *T. aestivum*–*D. villosum* introgression lines.

Thus, examples of E11 and E6 show that broad subgenomic or phylogenetic distribution of a repeat family does not preclude its cytogenetic informativeness. The diagnostic value of a FISH marker may be determined not only by its degree of genomic specificity, but also by characteristic local accumulation of repeat sequences and the reproducibility of the pattern in a specific cytogenetic context.

From an applied perspective, the repeat families identified here may provide additional cytogenetic tools for the characterization of Triticeae breeding material. After validation across additional accessions and genomic backgrounds, repeats showing pronounced subgenomic association could be used in combination with established FISH probes to improve the identification of St-, H-, and Y-derived chromatin in interspecific hybrids and introgression materials. Such markers may facilitate the cytogenetic monitoring of alien chromosomes or chromosome segments during the development and selection of breeding lines involving wild Triticeae germplasm. In particular, E9 and E10 are candidates for further evaluation as markers of St- and Y-associated chromatin, respectively, whereas E12 and E16 may complement existing probes for the identification of H-associated chromosome regions. However, their practical use will require confirmation of pattern stability and specificity across genetically diverse material.

Taken together, comparisons with other Triticeae indicate that the repeat landscape of *E. tsukushiensis* combines both broadly retained and rapidly differentiating components. Homologous repeat families may persist across related genomic lineages, whereas their abundance and chromosomal organization can diverge substantially at the species, subgenome, and chromosome levels. Similar patterns reported in *Pseudoroegneria*, *Hordeum*, *Thinopyrum*, *Aegilops*, wheat, and other *Elymus* species support a mosaic, family-specific model of repetitive DNA evolution rather than a uniform genome-wide response. In *E. tsukushiensis*, the contrasting organization of E1–E16 may therefore reflect different evolutionary trajectories superimposed on the histories of the St-, H-, and Y-subgenomes. However, broader comparative sampling of *E. tsukushiensis* and related diploid and polyploid Triticeae will be required to distinguish ancestral repeat patterns from changes that occurred after allopolyploidization.

## 4. Materials and Methods

### 4.1. Plant Material

We used the perennial wild grass *E. tsukushiensis* W6 PI 283170 (2n = 42, StStHHYY), originating from Japan and provided by the USDA-ARS Germplasm Resources Information Network (GRIN).

### 4.2. Sequencing and Repeatomes Structure

Young leaves of *E. tsukushiensis* were frozen in liquid nitrogen. Genomic DNA was extracted using the CTAB method. DNA concentration was determined with a Qubit 4 fluorometer (Thermo Fisher Scientific, Waltham, MA, USA), and integrity and quality were assessed by electrophoresis in 0.8% agarose gels. A shotgun library was prepared from 25 ng of genomic DNA using the Swift 2S Turbo DNA Library Kit (Swift Bioscience, Ann Arbor, MI, USA) following the manufacturer’s protocol. The average fragment size was approximately 350 bp; libraries were dual-indexed using the Swift 2S Turbo Unique Dual Indexing Kit (Swift Biosciences, Ann Arbor, MI, USA). Library quality was preliminarily assessed by test sequencing on an Illumina MiSeq platform. Main paired-end sequencing was performed on an Illumina NextSeq (Illumina, Inc., San Diego, CA, USA) using the NextSeq 500/550 Mid Output Kit v2.5, producing 2 × 151 nt reads. For *E. tsukushiensis*, we obtained 2,272,796 read pairs.

Adapter sequences, reads with ambiguous nucleotides, and low-quality segments were removed using BBDuk (BBMap suite) from the BBMap package. Read quality before and after filtering was checked with FastQC v0.12.1. To avoid length-dependent biases, reads were trimmed to a fixed length of 100 nt. From the cleaned dataset, 2,000,000 paired-end reads were randomly selected and analyzed with RepeatExplorer2 and its integrated Tandem Repeat Analyzer (TAREAN) module.

Based on graph-based clustering, the proportions of major repeatome components of *E. tsukushiensis* were determined. The content of each class was calculated from the fraction of reads included in the corresponding RepeatExplorer2 clusters.

### 4.3. Fluorescence in Situ Hybridization (FISH)

Metaphase chromosome preparations were made from root meristems of *E. tsukushiensis* W6 PI 283170. Root tips were fixed, and cytological preparations were prepared from meristems according to the protocol of [60]. As probes, we used the monomer sequences of satellite repeats E1–E16 previously published [27]. Probes were obtained by PCR amplification of the corresponding sequences using specific primers and labeled with biotin-16-dUTP (Sileks, Moscow, Russia) or digoxigenin-11-dUTP (Roche, Mannheim, Germany). PCR conditions were: initial denaturation at 95 °C for 5 min, followed by 35 cycles of 95 °C for 30 s, annealing at 60 °C for 30 s, extension at 72 °C for 30 s, and a final extension at 72 °C for 10 min. PCR product size and homogeneity were verified by electrophoresis in 1.5% agarose gels; only amplicons of the expected size were used for subsequent labeling.

Localization of satellite repeats on chromosomes was determined by FISH according to the protocol described in [61]. Biotinylated probes were detected with streptavidin-Cy3 conjugates (Vector Laboratories, Peterborough, UK), and digoxigenin-labeled probes with anti-digoxigenin-FITC conjugates (Roche, Mannheim, Germany). After hybridization, chromosomes were counterstained with DAPI in Vectashield mounting medium (Vector Laboratories, Peterborough, UK).

Following visualization and recording of signals from the first FISH hybridization, the slides were used for sequential hybridization with probes for subgenome identification. Before re-hybridization, slides were washed sequentially in 2 × SSC for 5 min at room temperature, then in 2 × SSC for 10 min at 42 °C, and in 0.2 × SSC for 5 min at 37 °C. For subgenome identification, we performed re-hybridization on the same slides with the following cytogenetic probes: St_2_-80 and Oligo-HvCSR [19,62]. For St-subgenome chromosome identification, we used the repeated DNA sequence St_2_-80 published by Wang et al. [28]. In contrast to the original method that relied on cloned sequences, here we obtained the FISH probe directly by PCR amplification. To cover the St_2_-80 sequence evenly, we designed four pairs of specific primers (Table 3) located in different regions of the repeat.

Genomic DNA of *E. tsukushiensis* served as PCR template. Amplification with each primer pair was performed separately. All four PCR products were labeled with digoxigenin-11-dUTP, pooled, and used as a composite FISH probe. This approach allowed us to generate the probe without cloning steps.

Slides were analyzed using a DFC 9000 GTC fluorescence microscope (Leica, Wetzlar, Germany), and images were processed with Adobe Photoshop 2017.1.1 (Adobe Inc., San Jose, CA, USA). Chromosomal localization of each repeat was determined on at least ten metaphase spreads obtained from independently prepared root tip chromosome preparations of the same plant.

### 4.4. Comparative Analysis of Relative Copy Number and Chromosomal Localization of Similar Repetitive Sequences

To compare quantitative representation of repeat clusters with FISH mapping results, we used previously published qPCR-derived relative copy numbers for E1–E16 [27]. The comparative analysis included values for *E. tsukushiensis* as well as for selected diploid representatives of St- and H-genomes—*P. spicata*, *P. tauri*, and *H. vulgare*. We used log_10_-transformed relative copy numbers. No new qPCR measurements were performed in this study. Because of differences in ploidy level, the comparison of relative copy numbers was used to reveal comparative trends in repeat representation and was not interpreted as a direct quantitative estimate of the contribution of individual subgenomes.

Data on the similarity of E1–E16 sequences to previously characterized Triticeae repeats were obtained in our earlier study [27]. In the present work, for comparative characterization we used only those similar sequences for which chromosomal localization had been established in published sources. For these, we recorded taxonomic affiliation, genomic association, type of chromosomal localization, and corresponding literature references.

## 5. Conclusions

The results of this study support the hypothesis that satellite-repeat abundance and chromosomal organization represent related but non-equivalent characteristics of repetitive DNA organization in the allopolyploid genome of *E. tsukushiensis*. FISH mapping showed that the investigated E1–E16 families differ markedly in their chromosomal organization among the St-, H-, and Y-subgenomes. Their distribution covers a wide spectrum of patterns—from local accumulation of large arrays in a single subgenome to broad two- and multisubgenomic dispersal. Particularly pronounced subgenomic association of large FISH-detectable arrays was observed for E7 and E9 with the St-subgenome, E10 with the Y-subgenome, and E12 and E16 with the H-subgenome.

Comparison of FISH patterns with previously obtained qPCR data demonstrated that the quantitative abundance of a repeat family and its physical organization on chromosomes are complementary but not identical characteristics. In particular, high copy number can coexist with local accumulation on a limited number of chromosomes, as in the case of E9, whereas low overall abundance does not preclude the formation of reproducible cytogenetically distinguishable loci, as shown for E16. The case of E10 further illustrates that a statistical association of repeat copy number with a particular genomic component should not be directly interpreted as evidence of its physical localization in that subgenome without cytogenetic validation. Thus, the comparison of qPCR and FISH data supports our hypothesis that whole-genome quantitative abundance does not necessarily predict the subgenomic localization of the major chromosomal arrays of a repeat family.

Among the investigated repeats, E9, E10, E12, and E16 showed potential for further evaluation as subgenome-associated cytogenetic markers. Following validation across diverse genetic backgrounds, such markers may also be useful for cytogenetic identification and monitoring of St-, H-, and Y-derived chromatin in interspecific hybrids and introgression materials. In the studied accession, the major detectable arrays of E9 were restricted to the St-subgenome and those of E10 to the Y-subgenome, whereas E12 and E16 showed preferential association with the H-subgenome. However, because the present analysis was performed on a single *E. tsukushiensis* accession, these patterns should presently be regarded primarily as characteristics of the studied genotype rather than as fixed species-level features. Their intraspecific stability, inheritance, and broader diagnostic applicability require validation using biologically independent and representative sampling, including additional plants and accessions and, where relevant, material from different populations or geographic origins. Assessment of inheritance will additionally require analysis of progeny and/or meiotic transmission.

More generally, joint interpretation of quantitative and cytogenetic data provides complementary information on repeat abundance and chromosomal organization and thereby allows a more complete characterization of subgenomic differentiation of repetitive DNA in polyploid Triticeae. Further comparative analyses involving closely related diploid and polyploid Triticeae species are required to determine whether the observed subgenomic differences were inherited from ancestral genomic lineages or arose during subsequent evolution following allopolyploidization.

## Figures and Tables

**Figure 1:**
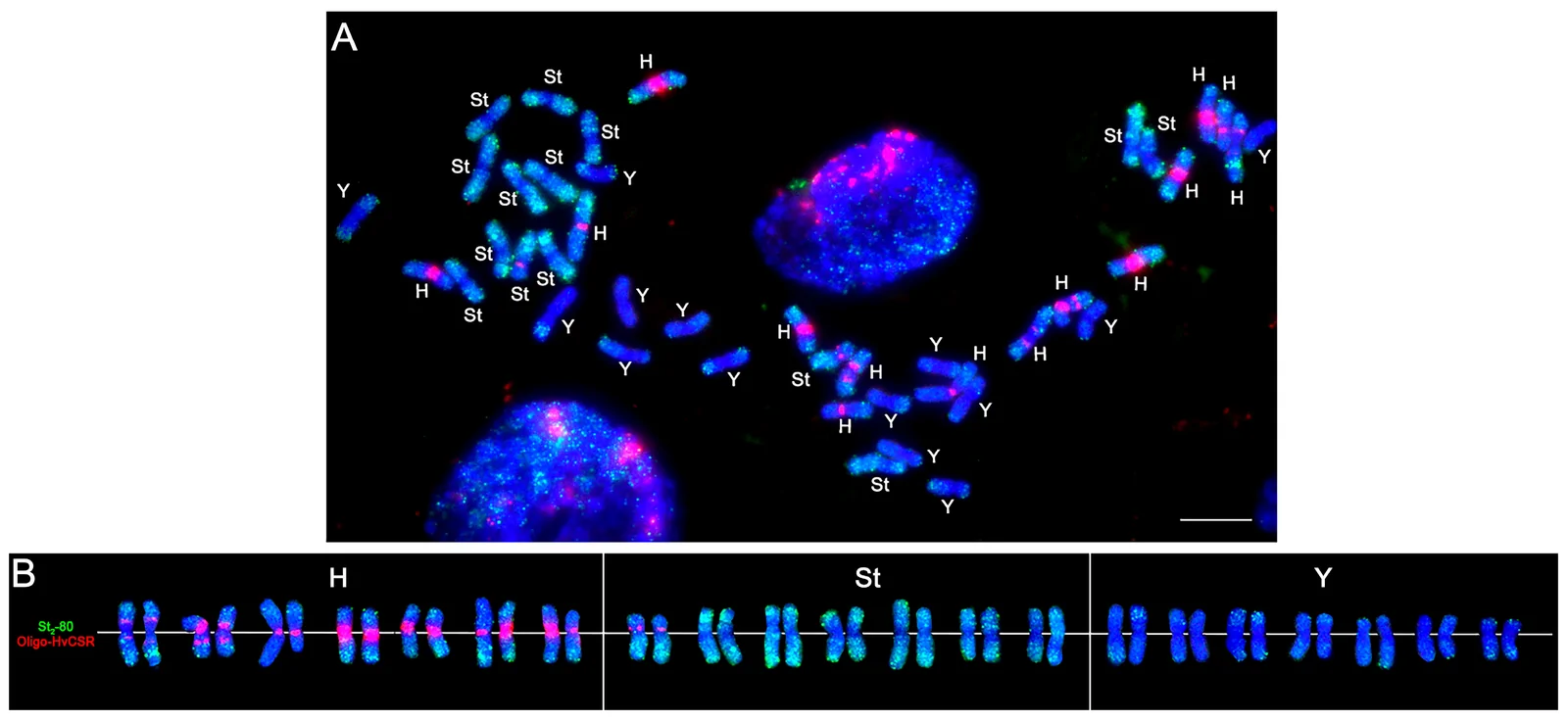
Differentiation of H-, St-, and Y-subgenomes of *E. tsukushiensis* by FISH using probes Oligo-HvCSR (red) and St_2_-80 (green): (**A**)—metaphase plate of *E. tsukushiensis* after fluorescence hybridization. (**B**)—karyogram showing chromosome distribution across H-, St-, and Y-subgenomes. Chromosomes counterstained with DAPI (blue). Bar = 10 µm.

**Figure 2:**
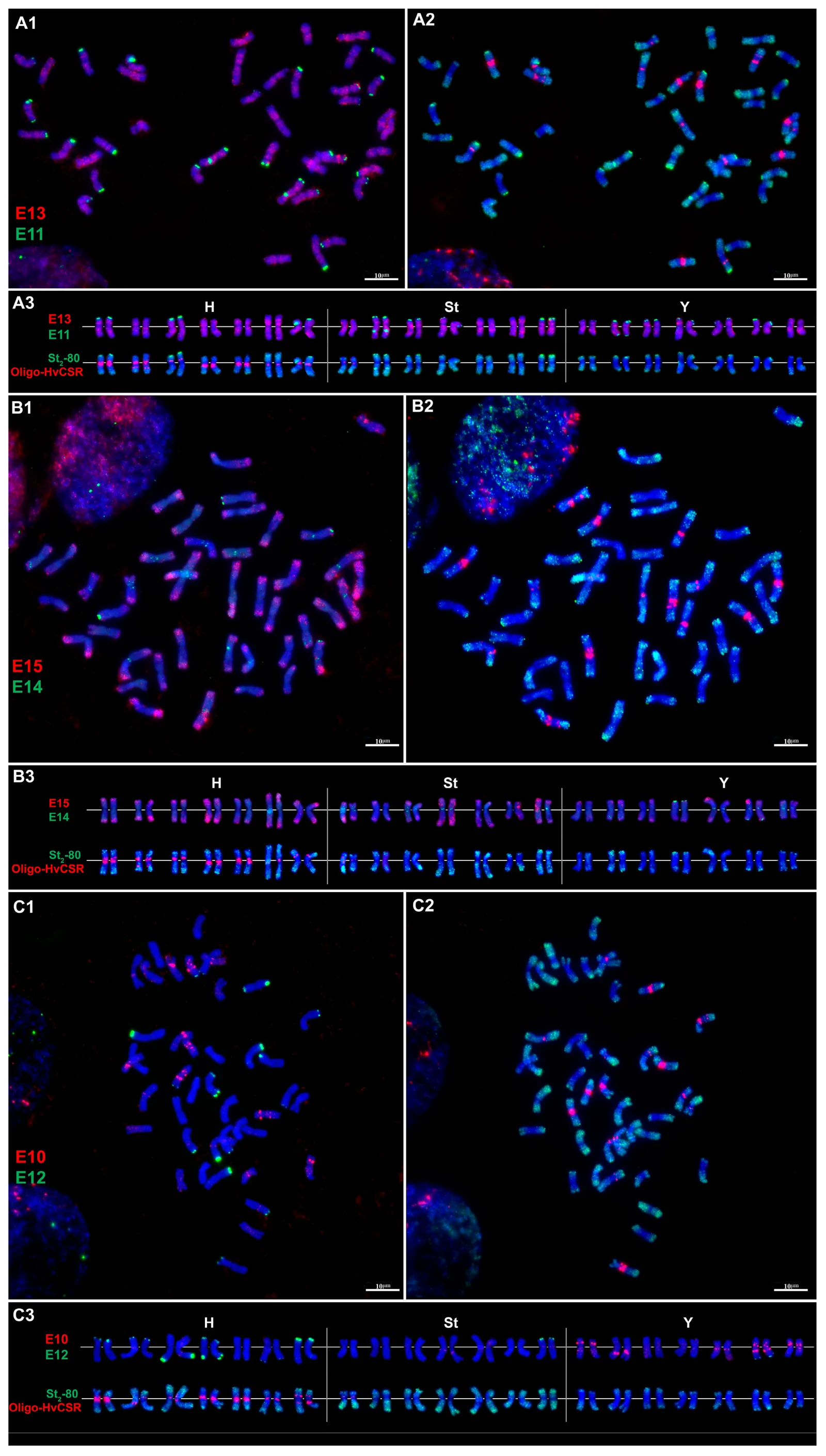
Localization of satellite repeat clusters followed by subgenome identification in *E. tsukushiensis*: (**A1**)—E13 (red) and E11 (green), (**B1**)—E15 (red) and E14 (green), (**C1**)—E10 (red) and E12 (green), (**A2**–**C2**)—Oligo-HvCSR (red) and St_2_-80 (green), and (**A3**–**C3**)—corresponding karyograms of *E. tsukushiensis* showing chromosome assignment to the H-, St-, and Y-subgenomes. Chromosomes counterstained with DAPI (blue). Bar = 10 µm.

**Figure 3:**
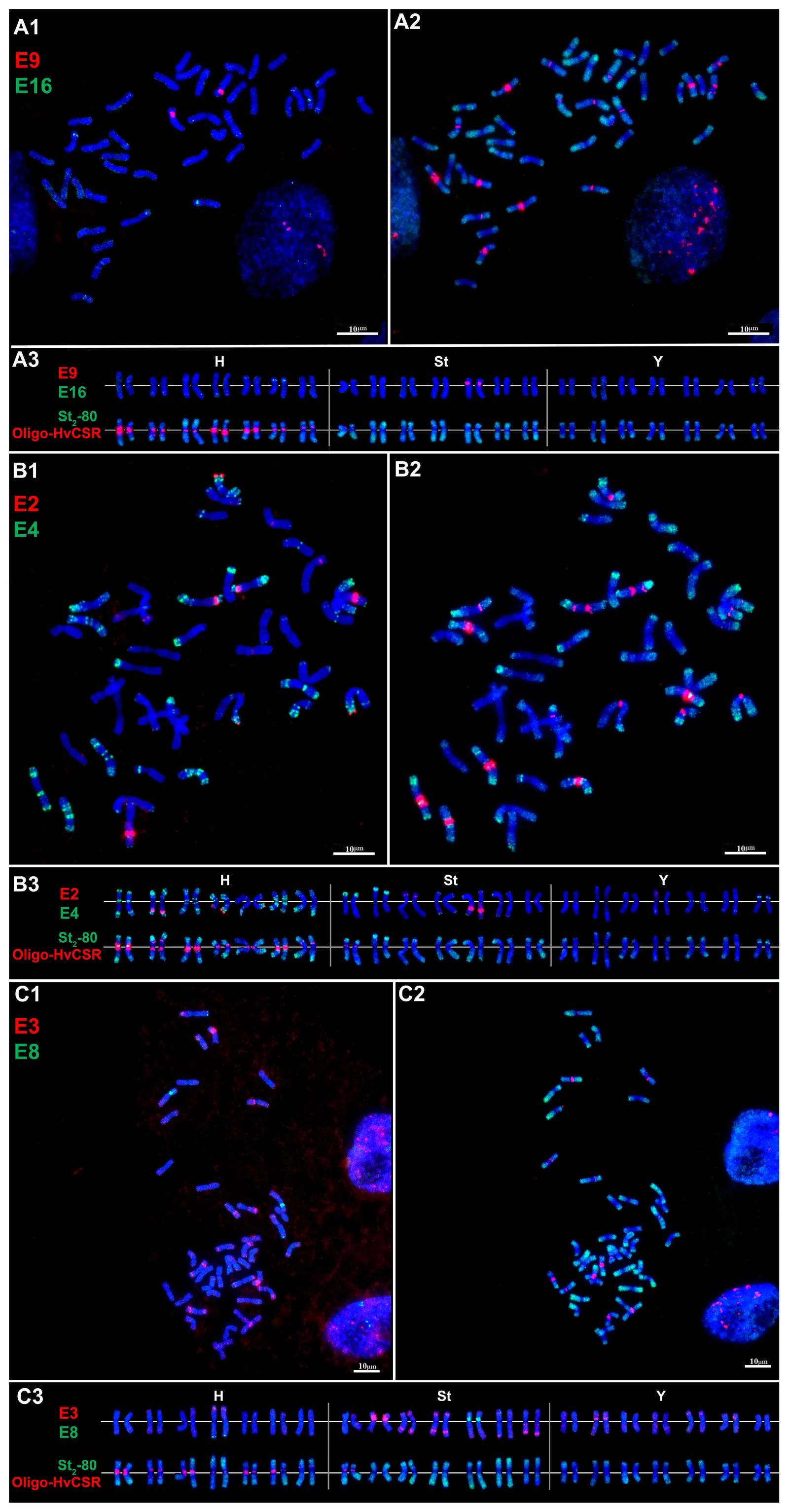
Localization of satellite repeat clusters followed by subgenome identification: (**A1**)—E9 (red) and E16 (green), (**B1**)—E2 (red) and E4 (green), (**C1**)—E3 (red) and E8 (green), and (**A2**–**C2**)—Oligo-HvCSR (red) and St_2_-80 (green). (**A3**–**C3**)—corresponding karyograms of *E. tsukushiensis* showing chromosome assignment to the H-, St-, and Y-subgenomes. Chromosomes counterstained with DAPI (blue). Bar = 10 µm.

**Figure 4:**
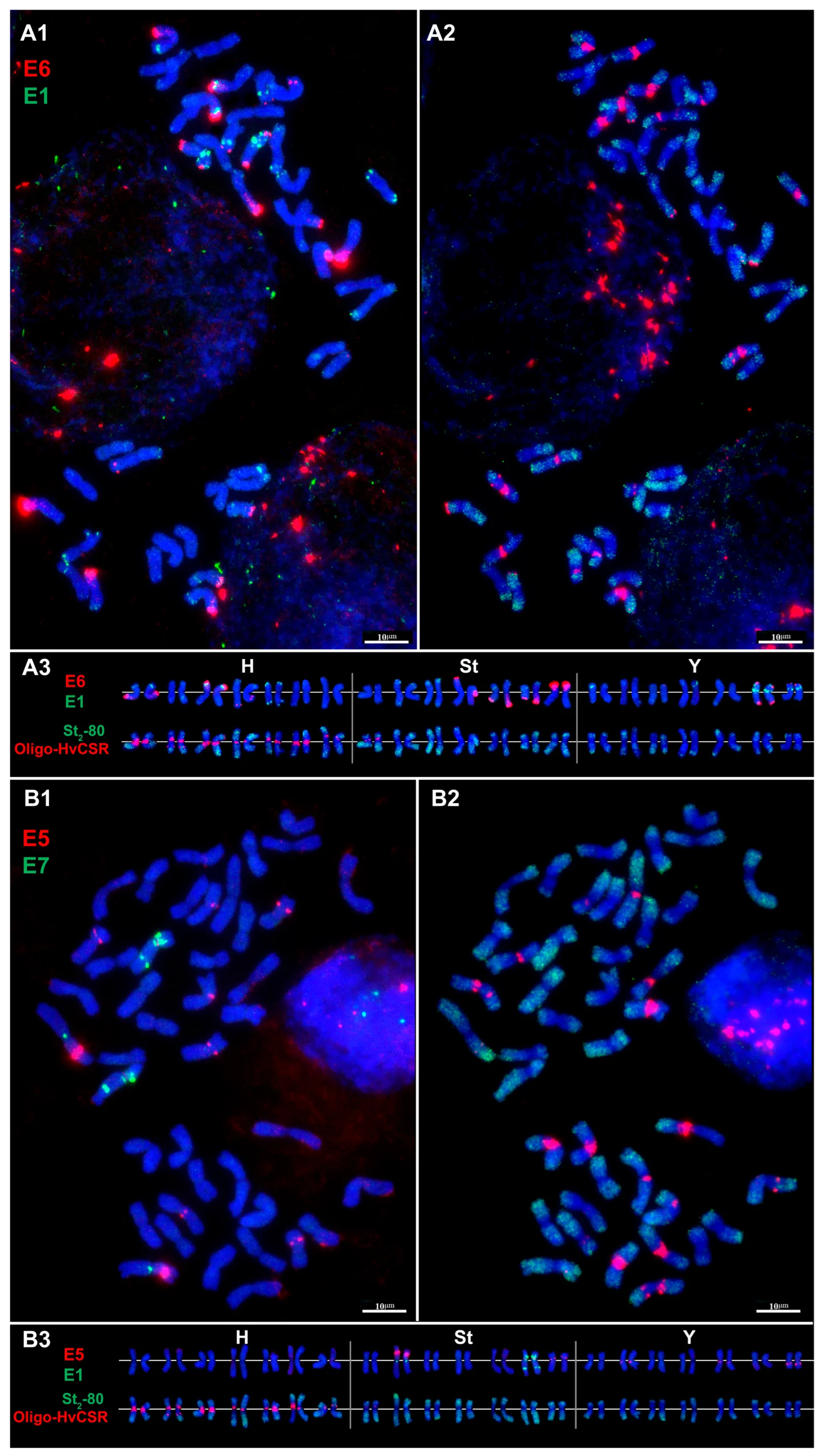
Localization of satellite repeat clusters followed by subgenome identification: (**A1**)—E6 (red) and E1 (green), (**B1**)—E5 (red) and E7 (green), (**A2**–**B2**)—Oligo-HvCSR (red) and St_2_-80 (green). (**A3**–**B3**)—corresponding karyograms of *E. tsukushiensis* showing chromosome assignment to the H-, St-, and Y-subgenomes. Chromosomes counterstained with DAPI (blue). Bar = 10 µm.

**Table 1:** Repeatome quantitative composition *E. tsukushiensis* based on RepeatExplorer2.

Type of DNA Repeat	Reads	Summarized %
Total repeatome		50.36
45S rDNA	6098	0.37
5S rDNA	1121	0.06
Satellite repeats	23,665	1.41
Mobile element		38.42
1. Class_I—RNA retroelements		36.87
1.1 Ty1/copia		13.4
Ale	844	0.05
Angela	158,453	9.41
Ikeros	1015	0.06
Ivana	78	0.01
SIRE	61,047	3.63
TAR	3546	0.21
Tork	379	0.03
1.2 Ty3/gypsy		23.47
Athila	175,725	10.45
Ogre	525	0.04
Retand	94,272	5.6
CRM	21,005	1.25
Tekay	103,268	6.13
2. Class_II—DNA transposons		1.55
EnSpm_CACTA	20,895	1.24
MuDR_Mutator	4402	0.26
PIF_Harbinger	700	0.04
Helitron	118	0.01
Unclassified and other repeats (residual)		10.1
plastid	13,895	-
mitochondria	117	-
Unclassified repeat (No evidence)	148,947	-

**Table 2:** Comparative relative copy numbers and chromosomal distribution of satellite repeats E1–E16 in *E. tsukushiensis*.

Repeat	*P. spicata*	*P. tauri*	*H. vulgare*	*E. tsukushiensis*	FISH-Positive Chromosome Pairs in *E. tsukushiensis* (H/St/Y)
E1	3.6	3.1	1.2	3.5	6/6/5
E2	4.0	4.0	2.1	4.1	3/3/0
E3	3.6	3.5	2.7	3.8	3/5/3
E4	4.8	4.8	4.5	5.8	7/5/3
E5	3.0	3.0	1.4	3.3	0/3/3
E6	4.5	6.2	−1.0	5.0	3/4/2
E7	3.6	3.2	−0.3	3.6	0/2/0
E8	1.8	1.6	−0.4	0.7	1/2/0
E9	2.0	2.4	−1.0	4.2	0/1/0
E10	1.2	1.8	1.5	3.5	0/0/5
E11	5.2	4.1	−0.4	4.5	3/4/6
E12	3.7	3.6	3.6	4.2	7/1/1
E13	3.9	4.1	2.9	3.2	7/7/7
E14	4.7	4.4	0.5	2.6	0/5/5
E15	4.0	4.2	2.9	4.0	7/7/7
E16	−0.4	−0.3	−0.7	−0.2	6/0/1

Relative copy numbers are presented as log10-transformed values obtained by qPCR in the previous study [27], and rounded to one decimal place. *P. spicata* and *P. tauri* were selected as representatives of St-genome lineages, and *H. vulgare* as a representative of an H-genome lineage; these species are not considered direct progenitors of the corresponding subgenomes of *E. tsukushiensis*. The H/St/Y column indicates the numbers of chromosome pairs with detectable FISH signals in the respective subgenomes of *E. tsukushiensis*. Detailed quantitative data, chromosomal localization patterns, and proposed subgenomic associations are provided in Table S2.

**Table 3:** Primers used for amplification of St_2_-80.

Repeat	Primers
St_2_-80 1	F: 5′-CCGATGGAAGGCATAGCAAC-3′ R: 5′-TGTTGATGTATAGAAGGCTGGTCA-3′
St_2_-80 2	F: 5′-TGAGCTAGACATTCCAGTCTTCTC-3′ R: 5′-GCTTCCATGAGACCCGAACT-3′
St_2_-80 3	F: 5′-AAGCACAAGAGTCCCATTATCCA-3′ R: 5′-CGCGTTTCCACCTTCCCTA-3′
St_2_-80 4	F: 5′-TGCGCTCCCACTCAAATCA-3′ R: 5′-TGATCGGCAAGTTGGCCT-3′

## Data Availability

The original contributions presented in this study are included in the article/Supplementary Material. Further inquiries can be directed to the corresponding author.

## Supplementary Materials

The following supporting information can be downloaded at: https://www.mdpi.com/article/10.3390/plants15213254/s1, Table S1: Chromosomal localization of previously characterized repetitive sequences similar to E1–E16 repeats; Table S2: Integrated quantitative and chromosome-level characteristics of E1–E16 repeats in *E. tsukushiensis*.
